# Effectiveness of Interventions to Improve Coping and Resilience of Frontline Mental Healthcare Professionals Towards Workplace Violence on Psychiatric Wards—A Systematic Review

**DOI:** 10.1111/inm.70016

**Published:** 2025-02-24

**Authors:** Paul Doedens, Laura M. Kiel‐Clayton, Joost G. Daams, Lieuwe de Haan

**Affiliations:** ^1^ Department of Psychiatry Amsterdam University Medical Center Amsterdam the Netherlands; ^2^ Urban Vitality–Centre of Expertise, Faculty of Health Amsterdam University of Applied Sciences Amsterdam the Netherlands; ^3^ Medical Library Amsterdam University Medical Center Amsterdam the Netherlands; ^4^ Arkin Amsterdam the Netherlands

**Keywords:** healthcare professionals, mental health care, psychiatry, resilience, systematic review, violence

## Abstract

Workplace violence (WPV) is a harmful phenomenon happening in psychiatric wards. Despite preventive efforts, mental health services cannot eliminate WPV. If mental health services can increase the coping and resilience of frontline mental healthcare professionals (FMHPs) towards WPV, it could contribute to their mental health and well‐being. To perform a systematic review of comparative studies on interventions to improve coping and resilience towards WPV aimed at FMHPs working in psychiatric wards. Systematic review on comparative intervention studies, with electronic searches in MEDLINE, Embase, Cochrane CENTRAL, PsycINFO and CINAHL. We registered our protocol in PROSPERO (CRD42022373757). Performing a meta‐analysis seemed not to be feasible, so we provided a narrative summary of the included studies, methodological quality and results. We included nine studies, with interventions focused on positive behavioural support, resilience enhancement and aggression management training. Most studies reported positive effects, though with a moderate to high risk of bias. Positive behavioural support, biofeedback and aggression management training are promising interventions in our review. Biofeedback interventions and positive behavioural support could be valuable additions to existing training programmes to improve coping and resilience. Future studies should focus on demonstrating the robustness of effects, the mechanism of increasing coping and resilience regarding WPV and the development and implementation of effective interventions.

## 1 Introduction

Nurses, social workers, and healthcare assistants work in the frontline of clinical mental healthcare. These frontline mental healthcare professionals (FMHPs) typically work in shifts on (inpatient) psychiatric wards and have intensive interaction with patients, family members and visitors. We refer to psychiatric wards as clinical units designed for the treatment and care of people with psychiatric illnesses situated in mental health services, hospitals, or other care institutions. Unfortunately, FMHPs have a high risk of encountering workplace violence (WPV) (Babiarczyk et al. 2020; Li et al. 2020; Liu et al. 2019; Mento et al. 2020; Nyberg et al. 2021; Ramzi et al. 2022; Ricoy‐Cano et al. 2024). The International Labour Organisation defines WPV as any action, incident or behaviour that departs from reasonable conduct in which a person is threatened, harmed or injured in the course of, or as a direct result of, their work (ILO 2003). Estimates of the lifetime prevalence of WPV against healthcare workers range from 34% to 79%, with nurses working in psychiatric wards being the professionals most impacted by WPV (Rossi et al. 2023). The prevalence of WPV might even be higher than reported due to underreporting (Spencer et al. 2023). WPV may affect FMHP's ability to provide mental healthcare and can result in professionals suffering from burnout, depression or post‐traumatic stress disorder (Flood et al. 2008; Hilton et al. 2022; Itzhaki et al. 2015; López‐López et al. 2019; Needham et al. 2005; Schuster and Dwyer 2020). In addition, healthcare professionals' intention to leave their current jobs increases due to WPV (Choi et al. 2023; Jiang et al. 2023; Park and Song 2023; Stafford et al. 2022; Zhao et al. 2024).

Research and policy about WPV focus primarily on identifying risk factors and the effectiveness of preventive interventions (Dack et al. 2013; Iozzino et al. 2015; Salzmann‐Erikson and Yifter 2020). There are attempts to manage WPV through interventions such as Safewards. Safewards is a programme that encourages staff members in psychiatric wards to cooperate with service users, family members and others involved in creating a safe and caring environment (Bowers 2014). However, no currently known interventions were able to prevent WPV in practice (Ward‐Stockham et al. 2022). FMHPs interact with patients suffering from a mental health crisis who feel unsafe or experience anxiety and paranoia. Subsequently, a mental health crisis is frequently associated with impaired impulse control and emotion regulation (Snyder et al. 2015). Therefore, despite all efforts for prediction and prevention, the management of conflict, anger and aggression in psychiatric wards will not lead to the total elimination of WPV from psychiatric wards. Therefore, FMHPs need to cope with WPV and its consequences.

In the case of stressful events, such as WPV, coping consists of cognitive and behavioural efforts to deal with internal or external demands, such as threat, harm or loss, created by the stressful situation (Lazarus and Folkman 1984; Carver 2013). Coping refers to the behavioural strategies used to manage stressful events and people's emotions related to these events (Folkman and Moskowitz 2004). Resilience is a concept related to coping and refers to a person's ability to adapt to the consequences of stressful events. Early definitions consider resilience as a narrow personal trait of maintaining mental health despite experiencing adversity (Herrman et al. 2011). Over time, the definition evolved to a more dynamic process in which an individual's resilience depends on time, context and type of adversity (Herrman et al. 2011). However, scientific literature omits a unified conceptualisation of the construct ‘resilience’ (Vella and Pai 2019). We use a definition in which resilience is the ability to recover from perceived adverse or changing situations through a dynamic adaptation process, influenced by personal characteristics, family and social resources and manifested by positive coping, control and integration (Caldeira and Timmins 2016).

The management of FMHPs' safety and security, and thereby their health, is a joint responsibility of employers, policymakers and society (Foster et al. 2019; Jacobowitz 2013; Shier et al. 2018; Spaan et al. 2023; Winstanley and Whittington 2002). It is not acceptable to transfer this responsibility to individual staff members. Nevertheless, improving FMHPs' coping and resilience may contribute to their well‐being if the prevention of WPV is not successful. Therefore, such safety management programmes should aim to enhance coping and resilience.

FMHPs' resilience is associated with better mental health, well‐being and protection against distress and burnout (Bui et al. 2023; Gao et al. 2017; Kinman and Grant 2016). Mental health services can improve the coping and resilience of FMHPs in psychiatric wards towards the inevitable occurrence of WPV, which could contribute to enhancing FMHPs' mental well‐being and reducing workplace violence‐related impacts.

Several authors recommend mindfulness or cognitive behavioural interventions to increase the resilience of FMHPs (Badu et al. 2020; Grant and Kinman 2012; Green and Kinchen 2021; Rushton et al. 2021). Breathing exercises are another intervention mentioned in the literature (Melnyk et al. 2020). These interventions help to increase resilience and stimulate effective coping with work stress. However, to help FMHPs with WPV, we probably need a more specific programme to support staff in such a demanding clinical environment (Dean et al. 2021).

The current systematic review provides an overview of comparative studies on effective interventions to improve FMHPs' coping and resilience towards WPV in psychiatric wards. By summarising the current state of evidence, we aim to give an overview of available interventions for clinical practice and identify gaps in the current evidence base on this subject.

### 1.1 Objective

To perform a systematic review of comparative studies on interventions to improve coping and resilience towards WPV aimed at FMHPs working in psychiatric wards.

## 2 Methods

### 2.1 Design

We performed a systematic review designed according to the PRISMA standards (Page et al. 2021). We registered this protocol in the PROSPERO‐register for systematic reviews (CRD42022373757).

### 2.2 Search Strategy

We used relevant literature and clinical experience to define keywords and synonyms. A medical information specialist (JGD) completed the search strategy and performed the electronic search in MEDLINE (Ovid), Embase (Ovid), PsycINFO (Ovid), Cochrane CENTRAL and CINAHL (EBSCOhost) from inception to February 2024. We elaborate on our complete electronic search strategy in Appendix A. In addition, we hand‐search reference lists of included manuscripts for additional studies. We employed no restrictions on language, publication date or publication status.

### 2.3 Study Selection

We selected manuscripts with the following inclusion criteria: (1) Randomised controlled trials (RCT) or other comparative, quantitative research designs (e.g., uncontrolled trials or before‐after designs); (2) Population of FMHPs (e.g., nurses) in psychiatric wards; (3) Intervention aimed at the improvement of (confidence in) coping or resilience towards WPV. Exclusion criteria are systematic reviews, conference contributions and interventions to prevent WPV and/or work stress. We included grey literature if the reference matched our selection criteria.

We mentioned our working definition of coping and resilience in the introduction section. The Confidence in Coping with Patient Aggression Instrument is a well‐known 10‐item scale in mental health research, developed by Thackrey (1987), which ranged from 10 to 50 points. This self‐assessment scale measures a professional's cognitive and behavioural ability to respond to aggressive behaviour. Confidence in coping is not the exact scope of this review. Still, we argue that the phenomenon of confidence in coping can serve as a proxy for their ability to cope with aggressive behaviour. Improving coping or resilience can be a study's primary or secondary aim for inclusion.

The first author performed an AI‐assisted first selection of the references in the online tool Rayyan (Khabsa et al. 2016). The AI tool suggested which references had a high probability of exclusion. The first author checked each suggestion of the AI tool for eligibility based on our in‐ and exclusion criteria and excluded obvious irrelevant references. Subsequently, two authors (PD and LMKC) performed an independent selection of the remaining manuscripts by title and abstract. Both authors assessed the full texts of the remaining references and settled disagreements through discussion. The last author (LdH) assisted in the decision‐making with unsolved differences.

### 2.4 Quality Assessment & Data Extraction

Two authors (PD & LMKC) performed data extraction based on a case record form. We extracted data from included manuscripts on the research design, population, intervention characteristics, comparative interventions, outcome measures, effect sizes, statistical precision, risk of bias and (potential) conflicts of interest. Two authors (PD and LMKC) performed the quality assessment of the included manuscripts using the Cochrane Collaboration's Risk of Bias Tools (RoB1) (Higgins et al. 2011). We described the methodological quality in our results section but did not exclude manuscripts based on low methodological quality. The risk of bias does, however, influenced the description of relevance in clinical practice of the reported interventions.

We assumed performing a meta‐analysis was not feasible due to high levels of (clinical) heterogeneity in the results. To structure the narrative syntheses of the evidence, we cluster the interventions in groups with similar objectives and content. We report the quantitative results of the studies. We use the interpretation suggested by Sawilowsky (2009) for effect sizes.

## 3 Results

### 3.1 Search & Selection Process

**FIGURE 1:**
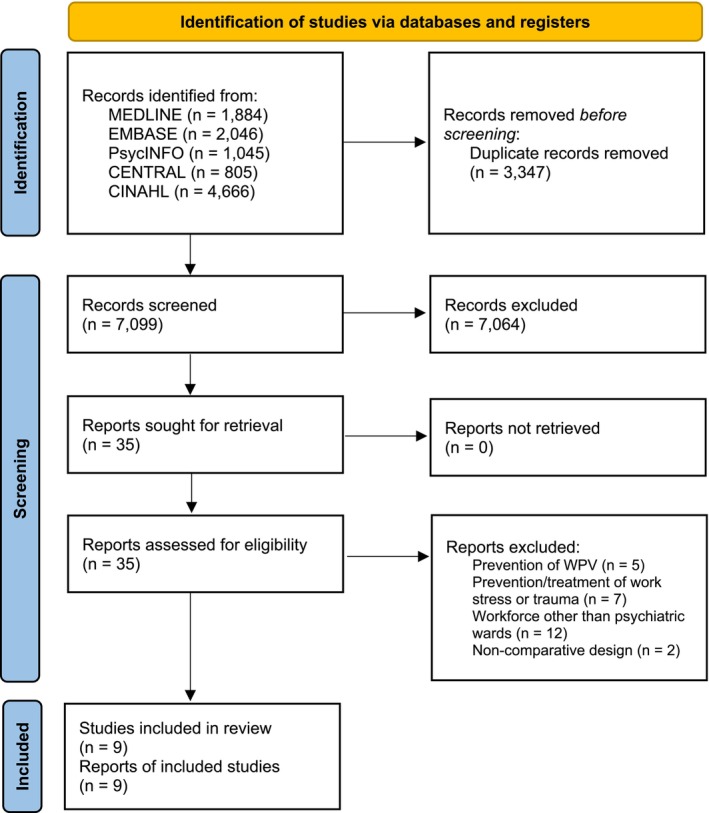
PRISMA flow chart.

We performed our search on 19 February 2024 (Appendix A), resulting in 7099 eligible manuscripts after removing duplicates. The first author removed 2061 references based on the AI‐assisted first screening. PD and LMKC performed independent selection based on the title and abstract of 5038 eligible manuscripts. After screening these titles and abstracts, 35 manuscripts were eligible for full‐text assessment. Eventually, we included nine manuscripts in our analysis. The PRISMA flow chart depicts the selection and screening process (Figure 1).

### 3.2 Summary of Included Studies

**FIGURE 2:**
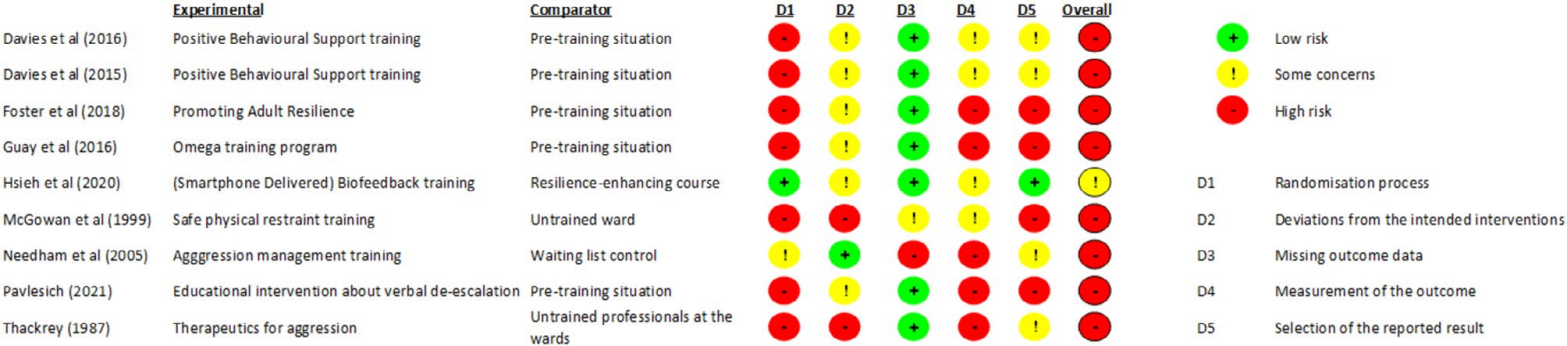
Risk of bias.

**FIGURE 3:**
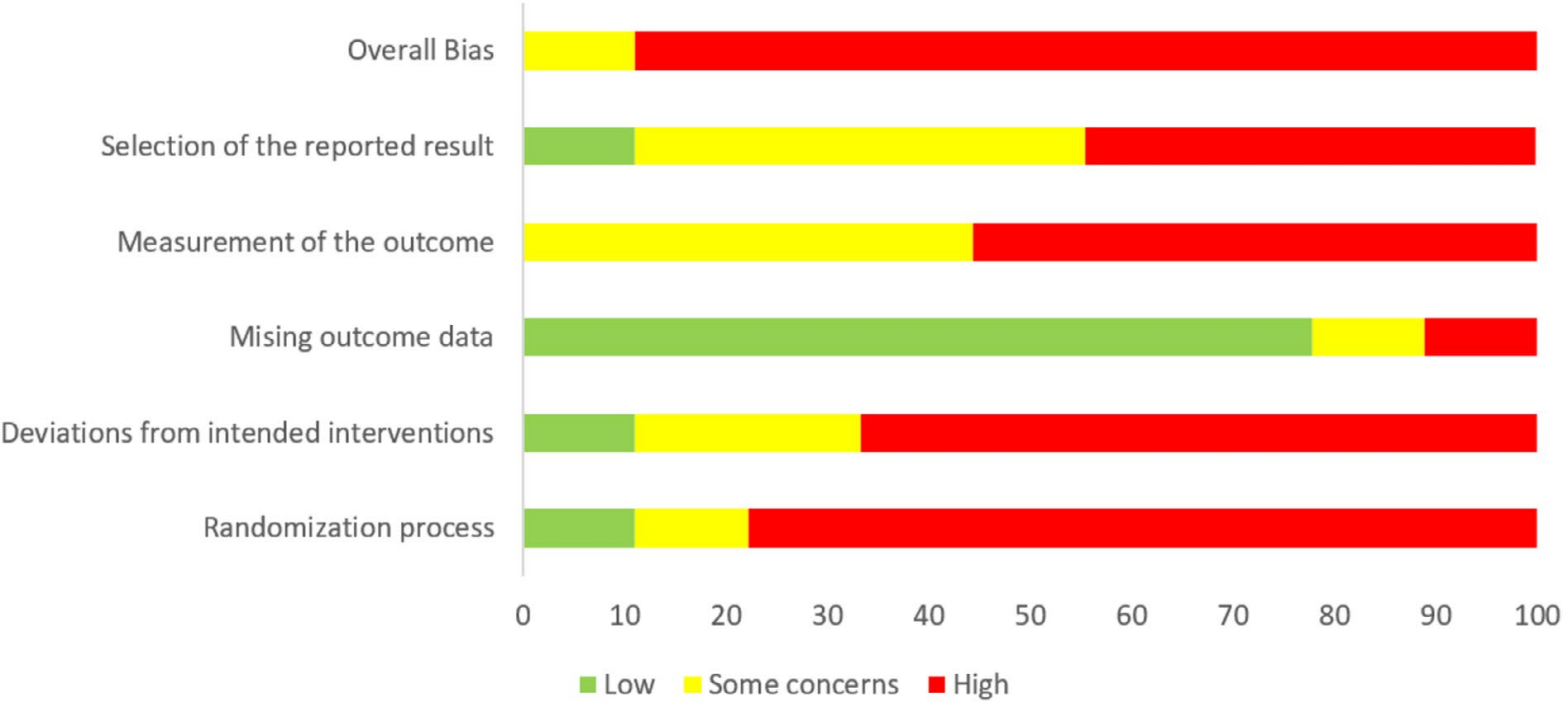
Risk of bias (as percentage).

Table 1 contains the details of the included studies. Five were before‐after studies (Davies et al. 2016; Davies et al. 2015; Foster et al. 2018; Guay et al. 2016; Pavlesich 2021), two were uncontrolled comparative studies (Mcgowan et al. 1999; Thackrey 1987), and two were randomised controlled trials (Hsieh et al. 2020; Needham et al. 2005a). The risk of bias in the included studies is moderate to high (Figures 2 and 3). The risk of bias in the before‐after studies is high by design, but both RCTs also have a moderate to high risk of bias due to the absence of blinding and high loss to follow‐up. Most studies used different interventions. Therefore, performing a meta‐analysis is not feasible, so we provide a narrative summary of the included studies, methodological quality, and results.

**TABLE 1:** Overview of included studies.

	Design	Country	Population and sample size	Intervention	Comparison	Outcome	Follow‐up
Davies et al. (2016)	Before‐after study	UK	Staff of medium secure forensic unit (*n* = 79)	Positive Behavioural Support training	Pre‐training situation	CCPAI, CHABA, CDS‐II	Directly post‐training
Davies et al. (2015)	Before‐after study	UK	Staff of medium secure forensic unit (*n* = 117)	Positive Behavioural Support training	Pre‐training situation	CCPAI, CHABA, CDS‐II	6 months
Foster et al. (2018)	Before‐after study	Australia	Staff of acute inpatient SMI‐units (*n* = 24)	Promoting Adult Resilience	Pre‐training situation	DASS‐21, SLS, RSPWB, Satisfaction with work, CSE, WRI	3 months
Guay et al. (2016)	Before‐after study	Canada	Staff on high‐risk psychiatric units (*n* = 89)	Omega training programme	Pre‐training situation	K6 scale, Exposure to violence, CCPAI	420 days
Hsieh et al. (2020)	Quasi‐RCT	Taiwan	Nurses of psychiatric wards (*n* = 159)	(Smartphone Delivered) Biofeedback training	Resilience‐enhancing course	CES‐D, OSI‐2, RS, HRV, respiration rate	6 weeks
Mcgowan et al. (1999)	Uncontrolled comparative study	Australia	Nurses in secure psychiatric intensive care units	Safe physical restraint training	Untrained ward	CCPAI	6 months
Needham et al. (2005)	RCT	Switzerland	Nurses of acute psychiatric wards (*n* = 114)	Aggression management training	Waiting list control	POAS‐S; TS, IMPACS	3 months
Pavlesich (2021)	Before‐after study	USA	Staff of mental health emergency department	Educational intervention about verbal de‐escalation	Pre‐training situation	CCPAI	90 days
Thackrey (1987)	Uncontrolled comparative study	USA	Professionals in community, inpatient and prison mental health	Therapeutics for aggression	Untrained professionals at the wards	CCPAI	18 months

Abbreviations: CCPAI = Confidence in Coping with Patient Aggression Instrument, CDS‐II = Causal Dimension Scale II, ÇES‐D = Centre for Epidemiologic Studies Depression scale, CHABA = Challenging Behaviour Attributions Scale, CSE = coping self‐efficacy, DASS‐21 = Depression, Anxiety & Stress Scale, HRV = heart rate variability, IMPACS = Impact of Patient Aggression on Carers Scale, OSI‐2 = Occupational Stress Indicator‐2, POAS‐S = Perception of Aggression Scale, RCT = randomised controlled trial, RS = Resilience Scale, RSPWB = Ryff's Scale of Psychological Well‐Being, SLS = Satisfactions with Life Scale, SMI = serious mental illness, TS = Tolerance Scale, UK = United Kingdom, USA = United States of America, WRI = Work Resilience Inventory.

We clustered the interventions into three groups with similar objectives and content. We described the interventions in Table 2. We described the results of the studies according to these three clusters. The first cluster consisted of two studies that trained staff members in Positive Behavioural Support. The second cluster included studies targeting staff members' resilience towards WPV. The third cluster consisted of studies that used training in aggression management to increase the (confidence in) coping of staff members with WPV.

**TABLE 2:** Description of interventions.

Cluster	Reference	Intervention	Description of intervention
1	Davies et al. (2016) &	Positive behavioural support training	PBS is a values‐led, multicomponent behavioural management framework, which serves as the theoretical basis for the training. Qualified staff members received a full day of training that covered basic knowledge on PBS and practicing skills (e.g., functional analysis and identifying prevention strategies). Unqualified staff members received half‐a‐day of training that covered basis knowledge on PBS and an introduction to antecedent, behaviour and consequence charts
	Davies et al. (2015)		
2	Foster et al. (2018)	Promoting adult resilience	An applied programme to enhance resilience, mental health and interpersonal communication skills with seven modules, delivered in weekly face‐to‐face meetings in a peer group by trained facilitators. Modules include understanding resilience, understanding and managing stress, challenging and changing negative self‐talk, drawing strength from adversity, promoting positive relationships, managing conflict and creating solutions for well‐being. Participants received e‐mail boosters between meetings and after the last meeting
	Hsieh et al. (2020)	(Smartphone delivered) biofeedback training	Biofeedback helps participants to recognise and, thereby, influence physiological responses to stress. All participants received a 2‐h resilience‐enhancing course. Biofeedback contained muscle relaxation techniques, several breathing exercises, and real‐time respiratory sinus arrhythmia biofeedback in weekly 60‐min sessions. The smartphone‐delivered group received a video every week for 6 weeks with shorter medication practices and processes of real‐time biofeedback
3	Guay et al. (2016)	Omega training programme	A 4‐day programme to improve coping skills and prevent aggressive behaviour by improving the knowledge, attitude and skills of the participant considering workplace violence. Peer trainers teach participants skills and interventions to adequately respond to aggressive behaviour to ensure safety of patients and staff. The training is based on the principles to protect yourself, assess the situation, predict behaviour, take time and to focus on the person with respect, professionalism, accountability and security
	Mcgowan et al. (1999)	Safe physical restraint training	A 1‐day training programme on legal and ethical issues, managing challenging behaviour, recognition and management of aggressive behaviour, physical restraint and breakaway techniques, in order to create a less restrictive environment
	Needham et al. (2005)	Aggression management training	A training programme, based on the work of Oud et al. (1997), with 20 lessons of 50 min in five consecutive days, providing knowledge, skills and techniques to the participants. Subjects of the lessons were types, causes and genesis of aggressive behaviour, reflections of own aggressive behaviour, conflict management, communication, prevention of aggressive behaviour, breakaway techniques and role‐play
	Pavlesich (2021)	Educational intervention about verbal de‐escalation	Educational class for frontline staff members on verbal de‐escalation techniques to decrease the rate of physical interventions and to improve the safety for healthcare workers. The training had no explicit theoretical framework from which it was developed
	Thackrey (1987)	Therapeutics for aggression	Two 4‐h sessions 1 week apart about legal and ethical issues, principles of psychological assessment, intervention techniques, staff teamwork and communication, physical methods for self‐protection and patient control in order to improve coping of nurses to increased confidence in their skills

### 3.3 Positive Behavioural Support‐Training

Davies et al. (2015) described a before‐after study on training in positive behavioural support (PBS) in a forensic medium secure service in the UK. PBS originates from learning disabilities institutions and is a multi‐component framework that includes understanding service users' behaviour, promoting service user involvement and preventive interventions for challenging behaviour. It provides professionals with risk assessment techniques and positive interventions to prevent or manage challenging behaviour, which includes WPV. Qualified staff members (*n* = 48) received a 1‐day training programme covering basic concepts of PBS and practical skills of using PBS in clinical practice. Unqualified staff (*n* = 31) received a 4‐h training covering basic concepts of PBS and an introduction to practical interventions concerning PBS. Qualified staff members were those team members who received professional training as a physician, nurse, occupational therapist, social worker or psychologist. Unqualified staff members had roles such as health support worker, activity coordinator or technician and had no formal training in healthcare. Outcome measures were the Confidence in Coping with Patient Aggression Instrument (Thackrey 1987) and the Challenging Behaviour Attributions scale (Hastings 1997). Participants received self‐assessment questionnaires at the start and end of the training. Unqualified staff had more confidence in coping with challenging behaviour at baseline (MD = 3.69, *p* < 0.007), but the post‐training measurement showed no meaningful difference (MD = 2.20, *p* = 0.164). Both groups (qualified staff MD = 7.28 and unqualified staff MD = 5.79) showed a substantial increase in confidence in coping after training (both *p* < 0.001).

Davies et al. (2016) used an almost identical method to replicate the previous study in a larger sample size (*n* = 117) in the United Kingdom. Furthermore, the authors introduced a follow‐up measurement 6 months post‐training, with identical outcome measurements at follow‐up. They reported similar findings in this replication study compared to Davies et al. (2015), and the results remained stable at the 6‐month follow‐up. Confidence in coping with challenging behaviour increased further in the qualified staff group after 6 months. Both studies had a high risk of bias due to the design (before‐after study), limited sample sizes, missing outcome data and reporting omissions (Figures 2 and 3).

### 3.4 Resilience‐Enhancing Interventions

Foster et al. (2018) described a before‐after study on the promoting adult resilience (PAR) programme after implementation in a group of mental health nurses in Australia. Staff members of two acute adult units received two full‐day workshops on the PAR programme, with follow‐up information e‐mails to boost the participant's knowledge afterwards. PAR aims to improve participants' resilience, mental health and well‐being and to decrease participants' interpersonal conflicts and stress. The programme's content integrates cognitive behavioural and interpersonal perspectives. The study performed outcome measures on mental health, well‐being, satisfaction with work, coping and resilience before the programme, immediately after and 3 months after completing the programme. The programme had positive effects (with minor to moderate effect sizes), measured immediately after the programme, on anxiety (ES = 0.36, *p* = 0.04), coping (self‐efficacy) (ES = 0.38, *p* < 0.01) and self‐regulatory: behavioural (a subscale of the resilience scale) (ES = 0.38, *p* = 0.03). Self‐regulatory is a subscale of the workplace resilience Inventory and measures behavioural self‐regulatory strategies (Mclarnon and Rothstein 2013). After 3 months, the programme had a moderate effect on stress (ES = 0.39, *p* = 0.02) but no substantial impact on resilience subscales and coping self‐efficacy, with effect sizes ranging from 0.03 (*p* = 0.86) to 0.18 (*p* = 0.31). This study had a high risk of bias due to the design (before‐after study), limited sample size and potential selective reporting of results.

Hsieh et al. (2020) described a quasi‐randomised controlled trial on the effects of (smartphone‐delivered) biofeedback training on depressive symptoms, work stress and resilience of psychiatric nurses in Taiwan. Biofeedback aims to assist professionals in recognising the physiological responses of their bodies and, eventually, to manipulate their physiology by using interventions such as breathing exercises. A frequently used form of biofeedback as a marker for stress is heart rate variability (HRV), which is the variation in time between heartbeats (Malik et al. 1996). Participants were nurses working in psychiatric wards who suffered from WPV and received a 2‐h resilience‐enhancing course. After this course, the first intervention group received biofeedback training in weekly one‐hour sessions for six consecutive weeks, which included, for example, breathing exercises and self‐guided muscle relaxation. The second intervention group also received biofeedback training and the resilience‐enhancing course, delivered by a smartphone app. Through this app, they received weekly videos on meditation exercises and real‐time biofeedback. The control group received no additional intervention besides the 2‐h resilience‐enhancing course. Outcome measures were depressive symptoms, occupational stress, HRV, respiration rate and resilience (Resilience Scale for Adults; range 29–203) (Friborg et al. 2003). The authors found no differences between the intervention groups and the control group regarding depressive symptoms, HRV, and respiration rate. Resilience increased compared to baseline within the biofeedback group (MD = 10.17, *p* < 0.001) and the smartphone‐delivered biofeedback group (MD = 15.64, *p* < 0.001), contrary to the control group (MD = 1.77, *p* = 0.321). Post hoc analysis of the between‐group differences showed no significant difference (*p* = 0.355) between the two intervention groups but significant differences between the intervention groups and the control group (*p* < 0.01). Occupational stress decreased within the smartphone‐delivered biofeedback (MD = −15,39, *p* < 0.001). Post hoc analysis supported this finding because of the significant difference with the control group (*p* = 0.005). The risk of bias in this study was unclear, primarily due to reporting omissions (Figures 2 and 3).

### 3.5 Aggression Management Training

Guay et al. (2016) described a before‐after study on the Omega education and training programme on high‐risk psychiatric units in Canada. Although the main objective of the 4‐day training programme is to minimise WPV towards professionals (*n* = 89) in mental health care, one of the secondary goals of the Omega programme is to improve confidence in coping with aggressive behaviour. Participants were nurses, orderlies, security agents and other staff members. The programme does not explicitly refer to resilience, but the secondary goal fits the definition used in this review. The study performed outcome measurements on psychological distress, exposure to violence and confidence in coping with aggressive behaviour. The Omega programme improved all outcome measures with small to medium effect sizes. Specifically for the outcome measure that fits our research objective, the authors reported an effect size d = −0.64 (*p* < 0.0001) for confidence in coping. This study had a high risk of bias due to the design (before‐after study), limited sample sizes, missing outcome data and potential selective reporting of results (Figures 2 and 3).

Mcgowan et al. (1999) described an uncontrolled comparative study between two previously trained secure psychiatric intensive care units in a 1‐day module for safe physical restraint and a similar unit that would receive training shortly. Participants (*n* = 70) were nurses working at the wards based in Australia. The training was part of a more extensive programme concerning safety. It included legal and ethical issues of restraint, effective communication with patients who show aggressive behaviour and physical restraint techniques. The primary outcome was the Confidence in Coping with Patient Aggression Instrument (Thackrey 1987). Before training, the nurses in the non‐trained ward scored lower on confidence (*M* = 2.64) than the trained nurses (*M* = 3.40). However, 6 months after training, the confidence of the trained nurses increased on all individual items (MD = 1.29, *p* < 0.01) and no longer showed any meaningful differences with the original wards. This study had a high risk of bias due to the design (before‐after study), limited sample size, self‐reported outcome data and potential selective reporting of results (Figures 2 and 3).

Needham et al. (2005a) described a multi‐centre randomised controlled trial on the effect of an aggression management training programme on perception, attitude and coping with aggressive behaviour. Three acute psychiatric wards in Switzerland received a 5‐day training programme, and three wards served as a waiting‐list control group. The training programme focused on topics like communication, de‐escalation, breakaway strategies, team techniques, workplace safety and reflection on the participants (*n* = 114 nurses) coping with aggressive behaviour. The Impact of Patient Aggression on Carers Scale (IMPACS) (Needham et al. 2005b) measured the relevant outcome. The scale measuring the impact focuses on adverse feelings after aggressive behaviour. An increase in adverse feelings may be a sign of deteriorating coping skills (Brown et al. 2005). At follow‐up, the authors found no differences between the groups. The risk of bias in this study was high due to the high loss to follow‐up (49%), a waiting list control group, and a limited sample size (Figures 2 and 3).

Pavlesich (2021) described a before‐after study on the feasibility of an educational intervention concerning verbal de‐escalation of aggressive behaviour on confidence in coping with aggressive behaviour. Professionals (*n* = 19) working at a mental health emergency department in the United States were eligible to participate in the study. The author performed outcome measurements 30, 60 and 90 days after training with the Confidence in Coping with Patient Aggression Instrument (Thackrey 1987). The confidence in coping with aggressive behaviour increased substantially (MD = 8.6). However, the author reported no inferential statistics, so whether this difference is statistically significant is unknown. There were no differences in follow‐up between groups of participants, such as nurses and patient care technicians. This study had a high risk of bias due to the design (before‐after study), limited sample size and potential selective reporting of results (Figures 2 and 3).

Thackrey (1987) described an uncontrolled comparative study between professionals who received aggression management training and those who did not (yet) on confidence in coping with aggressive behaviour. This was a secondary analysis in the developmental study of the Confidence in Coping with Patient Aggression Instrument. The professionals of interest (*n* = 106) worked at an inpatient psychiatric unit (*n* = 37), a community mental health organisation (*n* = 25) and a state psychiatric prison (*n* = 44) in the United States. Professionals who received training were compared to those who were unavailable but would receive training soon. Ward managers planned their staff in training based on schedules, vacation planning and working hours. The author measured the outcome 1 week and 18 months after training. Staff confidence increased after training compared to their untrained colleagues (MD = 14), and this difference remained stable at the follow‐up measurement (*p* < 0.001). This study had a high risk of bias due to the design (before‐after study), limited sample size, and reporting omissions (Figures 2 and 3).

## 4 Discussion

We found nine studies that matched our inclusion criteria, all with several methodological limitations resulting in a moderate to high risk of bias. Most studies (*n* = 7) had an uncontrolled comparative or before‐after design, which demands caution in interpreting the results. The included studies reported on eight different interventions, which we categorised into three clusters: (1) Positive Behavioural Support, (2) resilience‐enhancing interventions and (3) aggression management training. Based on these three clusters, we describe these interventions' scientific and practical context.

First, we found that PBS for staff members in medium secure forensic care improved their confidence in coping with challenging behaviour (Davies et al. 2016; Davies et al. 2015). PBS is a framework to assist staff members in understanding challenging behaviour based on a broad context in which the behaviour emerges (Gore et al. 2013). Evidence of effectiveness on patient outcomes is still limited, although studies report improvements in the quality of life of residents with developmental disabilities (Konstantinidou et al. 2023). The PBS framework consists of a functional assessment of the context in which the behaviour occurs, involvement of relatives, person‐centred planning of care, description of the specific behaviour and its triggers, preventive strategies, and focus on learning alternative behaviour (Gore et al. 2013). These subjects have common ground with interventional frameworks from mental health care related to WPV and prevention of coercive measures, such as Safewards (Bowers 2014) and Six Core Strategies (Lebel et al. 2014).

Second, we found evidence about two resilience‐enhancing interventions, the PAR programme and biofeedback‐assisted resilience training. The PAR programme showed no beneficial effect on the coping and resilience of staff members towards WPV (Foster et al. 2018). However, Hsieh et al. (2020) suggested that participants who received biofeedback support after resilience training improved regarding the outcome measure of resilience. This finding was independent of the platform for providing biofeedback, in person or by smartphone. Biofeedback is increasingly widespread in different sectors, including sports, education, business and healthcare. Witte et al. (2019) concluded that biofeedback is a promising component of stress management strategies. Lehrer et al. (2020) reported small to medium effect sizes on HRV biofeedback on emotional regulation, depressive symptoms and anxiety. Doody et al. (2021) reported that biofeedback interventions protected against stress and symptoms of post‐traumatic stress disorder at follow‐up (Doody et al. 2021; Maglione et al. 2022). Several (small) primary studies reported the positive effects of biofeedback interventions in laboratory settings (Hunter et al. 2019) and real‐life settings such as primary care (Orlando et al. 2021), art professionals (Brinkmann et al. 2020), medical students (Williams et al. 2020) and preservice teachers (Horgan et al. 2018). The abovementioned findings do not specifically focus on WPV; they show that biofeedback interventions can improve workplace settings on several outcome measures related to resilience and work stress.

Finally, we found several aggression management trainings that reported on (primarily) confidence in coping with aggressive behaviour. The only RCT in this matter showed no meaningful effects (Needham et al. 2005a). The other studies were uncontrolled and, therefore, highly susceptible to bias. The Omega programme, as described by Guay et al. (2016), is a training programme to teach skills in safety management and de‐escalation of aggressive behaviour, and it showed substantial effects on (among other outcomes) confidence in coping with aggressive behaviour. The training programmes described by different authors showed comparable content, although these studies gave less information on the training content (Mcgowan et al. 1999; Pavlesich 2021; Thackrey 1987). Training in aggression management and de‐escalation is one of the first strategies of health institutions and one of the first needs expressed by staff members who encounter aggressive behaviour. Several studies confirm that training can improve confidence in coping with aggressive behaviour in different populations of care professionals (Abozaid et al. 2022; Baig et al. 2018; Ferrara et al. 2017; Jones et al. 2023; Lamont and Brunero 2018). Training can also improve the attitude of professionals towards aggressive behaviour (Geoffrion et al. 2020). Whether training also prevents aggression from occurring remains questionable (Geoffrion et al. 2020; Spencer et al. 2018). Whether improved confidence in coping results in improvement of psychological coping after encountering aggressive behaviour is unknown. Also, a certain level of exposure to adversity or risk is deemed necessary to increase resilience (Vella and Pai 2019). If training specifically targets resilience, following up on trainees as they are exposed to adversity in clinical practice will be required. However, psychological coping and resilience are frequently mentioned omissions in most aggression management training programmes (Arbury et al. 2017; Farrell and Cubit 2005). Managing the emotional consequences of aggressive behaviour through training alone is a significant challenge (Heckemann et al. 2016). Other interventions to improve coping and resilience, such as counselling and peer support, might also be necessary. Therefore, although we found a clear indication for improvement in coping in several studies on aggression management training, future studies should focus on the association between confidence in coping, psychological coping, and resilience towards WPV.

## 5 Strengths & Limitations

This systematic review used rigorous methodology to provide an overview of the literature on a specific subject. However, the interpretation of our findings should incorporate some limitations.

First, our study aimed to find comparative intervention studies about interventions to improve coping and resilience towards WPV in psychiatric wards. Our search resulted in few studies suitable for inclusion. Second, the included studies were small and had several methodological limitations, resulting in a moderate to high risk of bias for all studies. Therefore, and because of heterogeneity, we did not perform a meta‐analysis. Although this is not a limitation by definition, it impacts our results for clinical practice. Thirdly, we did not perform a hand search to find studies published in peer‐reviewed journals that were not indexed in the central databases.

## 6 Conclusion

We found that Positive Behavioural Support, biofeedback‐assisted resilience training and aggression management training improved (confidence in) coping and resilience towards WPV. However, based on the methodological quality of the included studies, we are cautious to draw firm conclusions about these interventions. Besides methodological quality, the theoretical basis for the interventions is not clear enough in most cases. Whether this influenced the results of the studies is unknown. Most training programmes have the premise that improving the skills of staff members will enhance their resilience and coping. This theory is plausible but lacks a strong empirical basis. The description of future interventions should elaborate on their theoretical basis to give participants an explicit framework to interpret the intervention.

Nevertheless, we consider rigorous staff training in a clear aggression management framework and biofeedback combined with resilience training promising interventions that meet our objective. To assist in decision‐making, we need rigorous future studies with large sample sizes. Future studies and quality improvement projects could further investigate the influence of confidence in coping on resilience and explore the possibilities and feasibility of adding biofeedback interventions to existing training programmes to manage WPV in psychiatric wards.

### 6.1 Relevance for Clinical Practice

Coping with WPV in healthcare is challenging; no intervention would provide clinical practice with an ultimate solution. Many programmes for the management of WPV and its consequences, based on positive behavioural support or other frameworks, rely on the training of staff members in de‐escalation and safety procedures. These trainings have positive effects on confidence in coping with WPV in psychiatric care. However, whether this has a similar impact on resilience is still unknown. Furthermore, looking at alternative interventions besides training to support FMHPs is essential. Biofeedback could be such an intervention. Besides its effect on coping and resilience towards WPV, there is some evidence that biofeedback improves the capability to cope with stress and resilience in general. Mental health services must assist FMHPs in improving their coping and resilience towards WPV. They should provide professionals with sufficient training in aggression management and additional interventions to support and improve resilience and protect them from the consequences of WPV.

## Author Contributions

All authors listed meet the authorship criteria according to the latest guidelines of the International Committee of Medical Journal Editors. Paul Doedens and Lieuwe de Haan were involved in the conceptualisation and design of the manuscript. Joost G. Daams performed the systematic literature search. Paul Doedens and Laura M. Kiel‐Clayton performed the screening and analysis. Paul Doedens, Laura M. Kiel‐Clayton, Joost G. Daams and Lieuwe de Haan drafted, revised and finalised the manuscript.

## Conflicts of Interest

The authors declare no conflicts of interest.

## Data Availability

Data sharing is not applicable to this article as no new data were created or analyzed in this study.

## Appendix A

Search strategy

**Table:**

#	Ovid MEDLINE(R) ALL < 1946 to February 16, 2024 > Search date: 19 February 2024	Results
	Search	
1	(nurse? or nursing or ((health or care or hospital or psych*) adj3 (personnel or assistant? or worker? or staff))).ab,hw,jw,kf,ti. [frontline staff]	1 193 860
2	resilience, psychological/or “adaptation, psychological”/or emotional regulation/	114578
3	(resil?en* or adaptation or coping or (emotion* adj2 regulation?) or “self control”).ab,kf,ti.	370870
4	exp occupational stress/or “burnout, professional“/or”stress disorders, post traumatic”/	63343
5	(burnout or compassion fatigue or posttraumat* or post traumat* or ptsd or PTSS or distress or occupational stress or (emotion* adj2 (exhaust* or affect*))).ab,kf,ti.	272155
6	or/2‐5 [resilience]	688379
7	workplace violence/	1612
8	workplace/and (assault or violen* or aggress*).ab,kf,ti.	1720
9	(workplace violence or workplace stress or workplace resilience or workplace adversit*).ab,kf,ti.	2694
10	((assault or violen* or aggress*) adj10 (occupation* or profession* or work)).ab,kf,ti.	6276
11	(((assault* or violen* or aggress*) adj3 patient?) or (aggres* adj2 workplace)).ab,kf,ti.	14645
12	(occupational stressor? and (mental or psych*)).mp.	396
13	or/7‐12 [workplace violence]	23415
14	((assault or violen* or aggress* or (adverse adj2 event?)) and second victim?).ab,kf,ti.	187
15	and/1,6,13	1220
16	1 and 14	135
17	(resilienc? training and (stress management or smart)).ab,kf,ti. [stress management and resiliency training program ‐ SMART]	58
18	(resilience integration self‐awareness engagement or rise program).ab,kf,ti. [RISE program]	34
19	foryou.mp. [forYou program]	8
20	1 and ((Thackrey or Connor‐Davidson or Jalowiec) adj3 scale?).ab,kf,ti.	413
21	Current Experience Scale.ab,kf,ti.	2
22	Miller Behavioral Style Scale.ab,kf,ti.	33
23	or/15‐22	1884

#	Ovid Embase Classic + Embase < 1947 to 2024 February 16 > Search date: 19 February 2024	Results
	Search	
1	(nurse? or nursing or ((health or care or hospital or psych*) adj3 (personnel or assistant? or worker? or staff))).ab,hw,jw,kw,ti. [frontline staff]	1446709
2	psychological resilience/or psychological adjustment/or coping behavior/or emotion regulation/	94458
3	(resil?en* or adaptation or coping or (emotion* adj2 regulation?) or “self control”).ab,kw,ti.	437532
4	job stress/or burnout/or posttraumatic stress disorder/or compassion fatigue/	121774
5	(burnout or compassion fatigue or posttraumat* or post traumat* or ptsd or PTSS or distress or occupational stress or (emotion* adj2 (exhaust* or affect*))).ab,kw,ti.	369441
6	or/2‐5 [resilience]	835553
7	workplace violence/	2446
8	(workplace violence or workplace stress or workplace resilience or workplace adversit*).ab,kw,ti.	2964
9	((assault or violen* or aggress*) adj10 (occupation* or profession* or work)).ab,kw,ti.	7839
10	(((assault* or violen* or aggress*) adj3 patient?) or (aggres* adj2 workplace)).ab,kw,ti.	23101
11	(occupational stressor? and (mental or psych*)).mp.	397
12	or/7‐11 [workplace violence]	33324
13	((assault or violen* or aggress* or (adverse adj2 event?)) and second victim?).ab,kw,ti.	202
14	and/1,6,12	1292
15	1 and 13	135
16	(resilienc? training and (stress management or smart)).ab,kw,ti. [stress management and resiliency training program ‐ SMART]	85
17	(resilience integration self‐awareness engagement or rise program).ab,kw,ti. [RISE program]	67
18	foryou.mp. [forYou program]	13
19	1 and ((Thackrey or Connor‐Davidson or Jalowiec) adj3 scale?).ab,kw,ti.	431
20	Current Experience Scale.ab,kw,ti.	3
21	Miller Behavioral Style Scale.ab,kw,ti.	39
22	or/14‐21	2046

#	Ovid APA PsycInfo < 1806 to February Week 3 2024 > Search date: 19 February 2024	Results
	Search	
1	(nurse? or nursing or ((health or care or hospital or psych*) adj3 (personnel or assistant? or worker? or staff))).ab,hw,jw,id,ti. [frontline staff]	221892
2	resilience (Psychological)/or emotional adjustment/or exp emotional control/or coping behavior/	96896
3	(resil?en* or adaptation or coping or (emotion* adj2 regulation?) or “self control”).ab,id,ti.	237829
4	occupational stress/or posttraumatic stress disorder/or compassion fatigue/or emotional exhaustion/	67974
5	(burnout or compassion fatigue or posttraumat* or post traumat* or ptsd or PTSS or distress or occupational stress or (emotion* adj2 (exhaust* or affect*))).ab,id,ti.	171539
6	or/2‐5 [resilience]	409310
7	workplace violence/	1299
8	(workplace violence or workplace stress or workplace resilience or workplace adversit*).ab,id,ti.	1888
9	((assault or violen* or aggress*) adj10 (occupation* or profession* or work)).ab,id,ti.	7964
10	(((assault* or violen* or aggress*) adj3 patient?) or (aggres* adj2 workplace)).ab,id,ti.	4520
11	(occupational stressor? and (mental or psych*)).mp.	299
12	or/7‐11 [workplace violence]	13960
13	((assault or violen* or aggress* or (adverse adj2 event?)) and second victim?).ab,id,ti.	31
14	and/1,6,12	753
15	1 and 13	25
16	(resilienc? training and (stress management or smart)).ab,id,ti. [stress management and resiliency training program ‐ SMART]	28
17	(resilience integration self‐awareness engagement or rise program).ab,id,ti. [RISE program]	19
18	foryou.mp. [forYou program]	1
19	1 and ((Thackrey or Connor‐Davidson or Jalowiec) adj3 scale?).ab,id,ti.	165
20	Current Experience Scale.ab,id,ti.	0
21	Miller Behavioral Style Scale.ab,id,ti.	61
22	or/14‐21	1045

#	CRS: Cochrane Register of Studies Cochrane Central Register of Controlled Trials (CENTRAL) Search date: 19 February 2024	Results
	Search	
1	(nurse? or nursing or ((health or care or hospital or psych*) adj2 (personnel or assistant? or worker? or staff))):ti,ab,kw	
2	(resil?en* or adaptation or coping or (emotion* adj1 regulation?) or “self control”):ti,ab,kw	
3	(burnout or “compassion fatigue” or posttraumat* or post traumat* or ptsd or PTSS or distress or “occupational stress” or (emotion* NEAR/1 (exhaust* or affect*))):ti,ab,kw	
4	#2 or #3	
5	(workplace NEXT (violence or stress or resilience or adversit*)):ti,ab,kw	
6	((assault or violen* or aggress*) near/8 (occupation* or profession* or work)):ti,ab,kw	
7	(((assault* or violen* or aggress*) near/1 patient?) or (aggres* near/1 workplace)):ti,ab,kw	
8	(occupational stressor? and (mental or psych*)):ti,ab,kw	
9	#5 or #6 or #7 or #8	
10	#1 and #4 and #9	
11	((assault or violen* or aggress* or (adverse near/1 event?)) and second victim?):ti,ab,kw	
12	(resilienc? training and (“stress management” or smart)):ti,ab,kw	
13	(“resilience integration self‐awareness engagement” or “rise program”):ti,ab,kw	
14	foryou:ti,ab,kw	
15	((Thackrey or Connor‐Davidson or Jalowiec) NEAR/2 scale?):ti,ab,kw	
16	#1 AND (Current Experience Scale):ti,ab,kw	
17	(Miller Behavioral Style Scale):ti,ab,kw	
18	#10 or #11 or #12 or #13 or #14 or #15 or #16 or #17	805

#	CINAHL (Ebscohost) Search date: 19 February 2024	Results
	Search	
1	AB (nurse? or nursing or ((health or care or hospital or psych*) N2 (personnel or assistant? or worker? or staff))) OR SU (nurse? or nursing or ((health or care or hospital or psych*) N2 (personnel or assistant? or worker? or staff))) OR TI (nurse? or nursing or ((health or care or hospital or psych*) N2 (personnel or assistant? or worker? or staff)))	1117780
2	MH hardiness or MH “adaptation, psychological” or MH “emotional regulation”	55382
3	AB (resil?en* or adaptation or coping or (emotion* N1 regulation?) or “self control”) OR SU (resil?en* or adaptation or coping or (emotion* N1 regulation?) or “self control”) OR TI (resil?en* or adaptation or coping or (emotion* N1 regulation?) or “self control”)	166404
4	MH “stress, occupational” or MH “burnout, professional” or MH “stress disorders, post traumatic”	34005
5	AB (burnout or compassion fatigue or posttraumat* or post traumat* or ptsd or PTSS or distress or occupational stress or (emotion* N1 (exhaust* or affect*))) OR SU (burnout or compassion fatigue or posttraumat* or post traumat* or ptsd or PTSS or distress or occupational stress or (emotion* N1 (exhaust* or affect*))) OR TI (burnout or compassion fatigue or posttraumat* or post traumat* or ptsd or PTSS or distress or occupational stress or (emotion* N1 (exhaust* or affect*)))	152138
6	s2 or s3 or s4 or s5	301601
7	MH “workplace violence”	6757
8	MH “work environment” AND (AB (assault or violen* or aggress*) or SU (assault or violen* or aggress*) OR TI (assault or violen* or aggress*))	2000
9	AB (workplace violence or workplace stress or workplace resilience or workplace adversit*) OR SU (workplace violence or workplace stress or workplace resilience or workplace adversit*) OR TI (workplace violence or workplace stress or workplace resilience or workplace adversit*)	8618
10	AB ((assault or violen* or aggress*) N9 (occupation* or profession* or work)) OR SU ((assault or violen* or aggress*) N9 (occupation* or profession* or work)) OR TI ((assault or violen* or aggress*) N9 (occupation* or profession* or work))	4341
11	AB (((assault* or violen* or aggress*) N2 patient?) or (aggres* N1 workplace)) OR SU (((assault* or violen* or aggress*) N2 patient?) or (aggres* N1 workplace)) OR TI (((assault* or violen* or aggress*) N2 patient?) or (aggres* N1 workplace))	6907
12	AB (occupational stressor? and (mental or psych*)) OR SU (occupational stressor? and (mental or psych*)) OR TI (occupational stressor? and (mental or psych*))	163
13	S7 OR S8 OR S9 OR S10 OR S11 OR S12	17814
14	AB ((assault or violen* or aggress* or (adverse N1 event?)) and second victim?) OR SU ((assault or violen* or aggress* or (adverse N1 event?)) and second victim?) OR TI ((assault or violen* or aggress* or (adverse N1 event?)) and second victim?)	127
15	S1 AND S6 AND S13	1843
16	S1 AND S14	86
17	AB (resilienc? training and (stress management or smart)) OR SU (resilienc? training and (stress management or smart)) OR TI (resilienc? training and (stress management or smart))	13
18	AB (resilience integration self‐awareness engagement or rise program) OR SU (resilience integration self‐awareness engagement or rise program) OR TI (resilience integration self‐awareness engagement or rise program)	192
19	AB (foryou) OR SU (foryou) OR TI (foryou)	5
20	S1 and (AB ((Thackrey or Connor‐Davidson or Jalowiec) N2 scale?) OR SU ((Thackrey or Connor‐Davidson or Jalowiec) N2 scale?) OR TI ((Thackrey or Connor‐Davidson or Jalowiec) N2 scale?))	298
21	AB (Current Experience Scale) OR SU (Current Experience Scale) OR TI (Current Experience Scale)	9
22	AB (Miller Behavioral Style Scale) OR SU (Miller Behavioral Style Scale) OR TI (Miller Behavioral Style Scale)	27
23	S15 OR S16 OR S17 OR 18 OR S19 OR S20 OR S21 OR S22	4666
